# Physiological, environmental, and life-history drivers of haemosporidian infections in non-passerine birds from a rehabilitation center

**DOI:** 10.3389/fpara.2025.1568852

**Published:** 2025-08-04

**Authors:** Lis Marques de Carvalho e Vieira, Victor Aguiar de Souza Penha, Kevin J. McGraw, Amanda Vitória Dornelas da Silva, Erika Martins Braga

**Affiliations:** ^1^ Departamento de Parasitologia, Universidade Federal de Minas Gerais, Belo Horizonte, Minas Gerais, Brazil; ^2^ Department of Integrative Biology, Michigan State University, East Lansing, MI, United States

**Keywords:** blood parasites, plasmodium, haemoproteus, heterophil-to-lymphocyte ratio (H/L), parasitemia, microhematocrit, malaria

## Abstract

**Introduction:**

Pathogens and parasites play a crucial role in shaping ecological and evolutionary processes, influencing the behavior, physiology, and survival of their hosts across diverse ecosystems. Despite their taxonomic and functional diversity, non-passerine birds remain underrepresented in pathogen/parasite ecological research, providing an opportunity to explore how their unique life histories influence host-parasite dynamics. Investigating the susceptibility of non-passerines to infections, particularly in relation to physiological stress indicators such as heterophil-to-lymphocyte (H/L) ratios and microhematocrit levels, offers valuable insights into the complex interplay between health state, environmental conditions, and disease dynamics.

**Methods:**

We evaluated the occurrence of haemosporidian parasite (avian malaria) infections (Plasmodium spp. and Haemoproteus spp.) in individuals from six non-passerine bird species – Red-legged Seriema (Cariama cristata), Crested Caracara (Caracara plancus), Yellow-headed Caracara (*Daptrius chimachima*), Dusky-legged Guan (Penelope obscura), Gray-necked Wood-Rail (Aramides cajaneus), and Tropical Screech Owl (Megascops choliba) – that were admitted to the Wildlife Screening Center in Belo Horizonte, Minas Gerais, Brazil. We assessed whether blood-parasite infection occurrence was associated with hematological parameters (e.g. microhematocrit, H/L ratio), major injuries, age, body condition, season, co-occurrence of Trichomonas spp. infections, and presence of ectoparasites.

**Results:**

Of the 75 individuals analyzed, 37% were infected with haemosporidian parasites (Plasmodium spp. and Haemoproteus spp. combined). Age was a significant predictor of haemosporidian infection, with adults exhibiting higher overall haemosporidian parasite occurrence (both Plasmodium spp. and Haemoproteus spp. together), likely due to age-related chronic infection accumulation or higher mortality among infected juveniles. Also, individuals infected with Haemoproteus spp. only showed elevated H/L ratios, suggesting a physiological response to infection, and were more frequently infected during the rainy season, likely reflecting optimal vector conditions. No significant associations were observed between blood-parasite infection occurrence and other factors such as physical condition, major injuries, co-occurring Trichomonas spp., or the presence of ectoparasites.

**Discussion:**

These findings highlight the importance of considering physiological, environmental, and life-history factors when investigating malaria infections in non-passerine birds. By advancing our understanding of host-parasite interactions in these underrepresented species, this study contributes valuable knowledge to inform conservation, rehabilitation, and wildlife-management strategies for these less-studied birds.

## Introduction

Pathogens and parasites are fundamental drivers of host population dynamics, shaping behavior, physiology, and ecology across diverse ecosystems and host species (Woolhouse et al., 2002). Among these hosts, non-passerine birds stand out as a fascinating yet underexplored group in pathogen and parasite ecological research, with the potential to offer valuable insights into host-pathogen interactions across varied contexts (Santiago-Alarcon et al., 2014). Though comprising less than half of all bird species, non-passerine birds are generally large (with exceptions like hummingbirds) and include flightless runners (ratites) and swimmers (penguins), marshland waders (e.g. rails), and nocturnal hunters (owls), hence exposing them to a wide array of parasite and pathogen threats (Valkiunas, 2005; Gill, 2007).

Notably, non-passerines play a pivotal role in the dynamics of vector-borne diseases and the potential spillover of pathogens to other groups, including mammals (Valkiunas, 2005; Tsiodras et al., 2008). For example, when non-passerine species diversity is high, West Nile virus (WNV) transmission tends to decrease; this is because less competent hosts draw mosquitoes away from species that are better at amplifying the virus, which in turn reduces the risk of infection for other animals and humans (Ezenwa et al., 2006). Additionally, the foraging and nesting behaviors of non-passerines frequently bring them into contact with human-altered landscapes and livestock, thus increasing pathways for pathogen transmission. For example, waterfowl and shorebirds harbor avian influenza (Fenton et al., 2018) and coronaviruses, increasing the risk of spillover to farmed animals and humans (Rahman et al., 2021).

Among the many microbial threats to wild animals, haemosporidians represent a diverse group of vector-borne protozoan parasites that permit detailed examination of host susceptibilities, environmental selection pressures, and transmission dynamics. Haemosporidians are heteroxenous protozoa of broad ecological and veterinary importance, distributed worldwide except in Antarctica, and infecting various vertebrate hosts across different biomes (Valkiunas, 2005; Pacheco et al., 2011). In Brazil, they exhibit an average prevalence of ~28% in avian hosts (Ferreira-Junior et al., 2018; Oliveira et al., 2020; Fecchio et al., 2021c, Fecchio et al., 2021b, Fecchio et al., 2022; da Silva Rodrigues et al., 2021; de Angeli Dutra et al., 2023). These malaria parasites shape ecological dynamics by altering host physiology, behavior, and immune function, which in turn influence host interactions with vectors, conspecifics, and other species within their microenvironment and broader ecosystems (Carlson et al., 2015; Poulin, 2021). Although transmission dynamics have been well-studied in Passeriformes, research has primarily focused on their role in the spillover of virulent haemosporidian strains to naïve hosts via migration or introduction (Dinhopl et al., 2015; Niebuhr et al., 2016; Muriel et al., 2021), the crossing of wild-domestic boundaries (Ferreira-Junior et al., 2018), and interactions among unlikely host populations in rehabilitation centers and zoos (Sijbranda et al., 2017; Verwey et al., 2018; Ortiz-Catedral et al., 2019; Yoshimoto et al., 2021; González-Olvera et al., 2022). In contrast, much less is known about haemosporidian parasite transmission dynamics involving non-passerine birds. These interactions highlight the need to understand what makes non-passerine birds susceptible to disease, given their critical role in shaping disease dynamics and potential spillover to other animals. Given the diversity of non-passerines, it is likely that the risk and progression of haemosporidian infections in wild non-passerines are shaped by a multifaceted combination of biological, environmental, and anthropogenic factors.

Within avian communities, the impact of pathogens/parasites on hosts can be strongly influenced by age, with juveniles being particularly susceptible due to their relatively naïve immune systems or differences in behavior that may alter pathogen exposure (Wilson et al., 2002). For instance, low pathogenic influenza has been shown to cause greater severity in juvenile ostriches (*Struthio camelus*), primarily due to their limited capacity to immunologically cope with the virus (Capua et al., 2000). Similarly, juvenile Galapagos penguins (*Spheniscus mendiculus*) exhibit lower microbiome alpha diversity compared to adults, a difference likely driven by age-related behavioral patterns and potentially associated with increased susceptibility to pathogenic infections (Rohrer et al., 2023). Beyond age, an individual’s overall physical condition also plays an important role in infection risk. Birds in otherwise poor health may allocate fewer resources to immune defenses, increasing their susceptibility to infections and impairing their ability to manage pathogen burdens (da Silva Rodrigues et al., 2021). In American crows (*Corvus brachyrhynchos*), for example, poor body condition was related to a greater predisposition to several diseases (e.g., poxviral dermatitis, fungal pneumonia, enteritis) and consequent mortality (Townsend et al., 2010). Haemosporidian parasites have also been linked to decreased body condition in house martins (*Delichon urbica*; Marzal et al., 2013). However, it remains unclear whether individuals in poorer condition are more susceptible to infection, or if infection itself leads to declines in host condition.

Seasonal environmental fluctuations can further shape infection dynamics in wild birds, as changes in temperature, precipitation, and vector abundance can influence both host susceptibility and parasite transmission rates. Seasonality plays a crucial role in the transmission dynamics of avian malaria parasites, including *Plasmodium*, *Haemoproteus*, and *Leucocytozoon* (Valkiunas, 2005). In many regions, infection prevalence and parasite diversity fluctuate between the rainy and dry seasons, largely due to vector availability (Schultz et al., 2011; Hernández-Lara et al., 2017; Haghtalab et al., 2020; Fecchio et al., 2021a). The rainy season typically fosters higher transmission rates, as mosquito and biting midge populations—key vectors of *Plasmodium* and *Haemoproteus*, respectively—proliferate in response to increased humidity and water availability (Colombo and Joly, 2010; Tchoumbou et al., 2020). This seasonal peak in vector abundance often leads to a rise in infection prevalence and a higher likelihood of new infections, though the specific patterns depend on the haemosporidian parasite genus. For instance, *Leucocytozoon* spp. infections have been shown to increase with precipitation, likely due to the favorable conditions for black fly vectors, whereas *Plasmodium* spp. prevalence decreased overall in a global study, suggesting more complex seasonal drivers influencing its transmission in birds (Fecchio et al., 2021a).

Measurement of other physiological indicators, such as heterophil-to-lymphocyte ratios (H/L) and microhematocrit levels, can provide deeper insights into an individual’s stress, immune function, and overall health, all of which influence their infection susceptibilities and disease dynamics (Ricklefs and Sheldon, 2007; Satyaningtijas et al., 2020). Elevated H/L ratios are commonly associated with chronic stress and reduced immune function, making individuals more susceptible to malaria. For example, owls (from six different species) that were infected with *Leucocytozoon* spp. had higher H/L ratio compared to uninfected individuals, suggesting that individuals under higher stress are more susceptible to *Leucocytozoon* infection, or infection with *Leucocytozoon* depleted B-cells, rapidly decreasing lymphocyte levels compared to heterophils (Martín-Maldonado et al., 2023). Also, higher H/L ratios were associated with higher haemosporidian parasitemia in a diverse group of five Brazilian bird species (Rodrigues et al., 2021). In addition, low microhematocrit levels, indicative of anemia or poor oxygen transport capacity, further compromise a bird’s overall condition and ability to combat infections (Mitchell and Johns, 2008). Anemia is a clinical condition that has been consistently found to be associated with the acute phase of malaria infection (Kocan, 1968; Valkiunas, 2005).

Here we investigated several environmental, physiological, and life-history predictors of haemosporidian parasite infections in several species of non-passerine birds from Brazil. Non-passerine birds are less frequently studied in the context of avian malaria and other related haemosporidian parasites compared to passerines, but research indicates that they can serve as both competent and incidental hosts for *Plasmodium* spp. parasites, with varying susceptibility across taxa (Bell et al., 2020; Ilgūnas et al., 2022; Lotta-Arévalo et al., 2023). Some groups, such as pigeons and doves (Columbiformes), appear to be relatively resistant to infections, while others, like raptors and shorebirds, exhibit a broader range of susceptibilities (Santiago-Alarcon et al., 2014; Soares et al., 2016b; Capasso et al., 2023). Differences in host competence may be influenced by immune defenses, life-history traits, and habitat use, as many non-passerines occupy ecological niches that may limit exposure to mosquito vectors (e.g., marine environments, high-altitude zones, nocturnal habitats; Valkiunas, 2005; Santiago-Alarcon et al., 2014). Additionally, studies suggest that certain avian malaria lineages exhibit host specificity, with some *Plasmodium* spp. rarely detected in non-passerines (Lotta-Arévalo et al., 2023; Capasso et al., 2023). Despite these insights, significant knowledge gaps remain regarding the transmission dynamics, pathological effects, and potential evolutionary consequences of avian malaria in non-passerine hosts, highlighting the need for further research, especially in tropical and highly anthropogenically modified environments.

The objective of the present study was to evaluate the occurrence and severity of a common blood parasite – haemosporidians (Haemosporida, genera *Plasmodium* and *Haemoproteus*) – in six different non-passerine bird species from Brazil: red-legged seriema (*Cariama cristata*), crested caracara (*Caracara plancus*), yellow-headed caracara (*Daptrius chimachima*), dusky-legged guan (*Penelope obscura*), gray-necked wood-rail (*Aramides cajaneus*), and tropical screech owl (*Megascops choliba*). We studied individuals that were taken into the Wildlife Screening Center from the Brazilian Institute of Environment and Renewable Natural Resources (IBAMA) in Belo Horizonte, Minas Gerais, Brazil. We tested whether parasite occurrence and severity were related to hematological parameters (H/L ratio and microhematocrit levels), presence/absence of major injuries (burns, bone displacements, and fractures), age (juvenile vs. adults), body condition upon arrival at the rehabilitation center (poor vs. good), season (rainy vs. dry), as well as the occurrence of trichomoniasis (Order Trichomonadida, genus *Trichomonas* spp.), and ectoparasites (ticks and mites). We predicted that individuals with poor hematological parameters (high H/L ratio, low microhematocrit levels), major injuries, poor arrival condition, of juvenile age, sampled during the rainy season, and also infected with trichomoniasis and ectoparasites will have the highest haemosporidian parasite occurrences and severities. Overall, developing this deeper understanding of malaria parasites in these non-passerines can inform conservation efforts, improve rehabilitation practices, and enhance knowledge of host-parasite dynamics in diverse, tropical avian species.

## Methods

### Data collection

**Figure 1:**
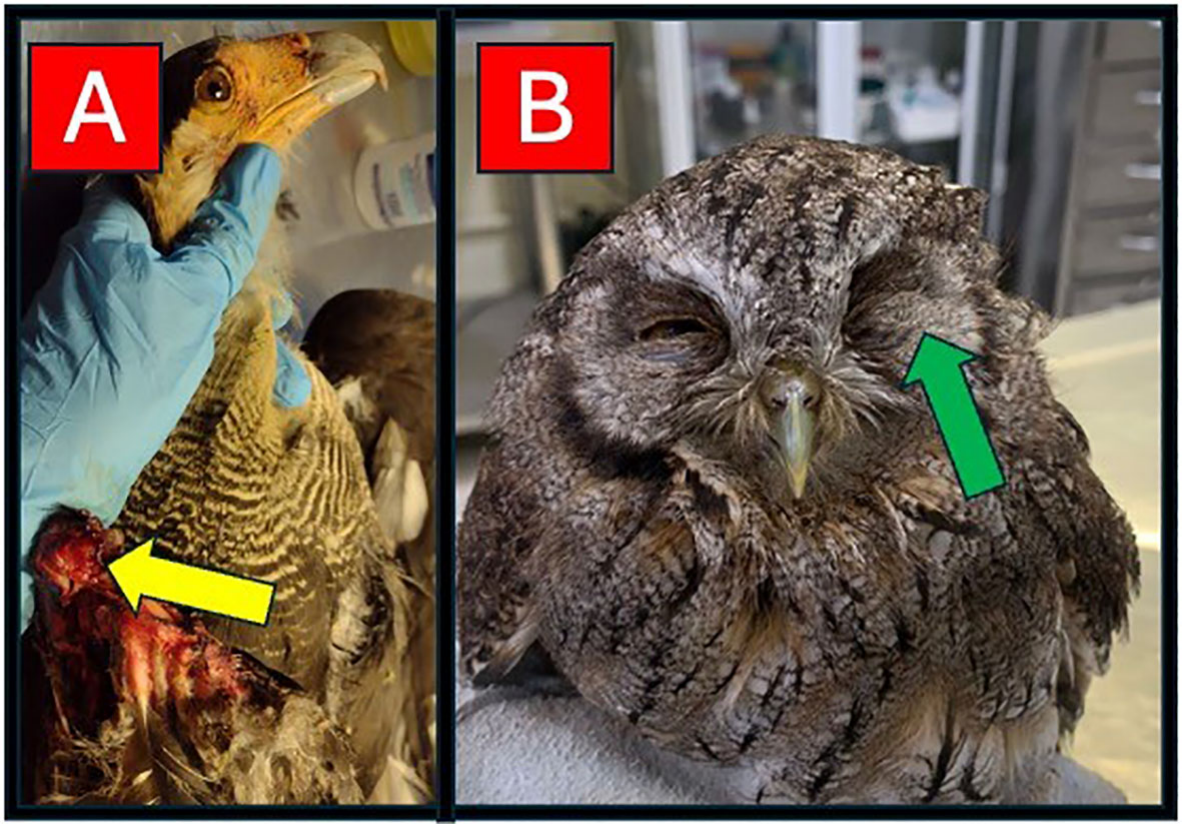
Selected images of birds taken into the screening center, with examples of different injuries. **(A)** Crested caracara (*Caracara plancus*) with a major injury on the right wing (yellow arrow); and **(B)** tropical screech-owl (*Megascops choliba*) with a major injury on the left eye (green arrow).

We sampled individuals received at the Wildlife Screening Center (Centro de Triagem de Animais Silvestres – CETAS-BH) from the Brazilian Institute of Environment and Renewable Natural Resources (IBAMA) in Belo Horizonte, Minas Gerais, Brazil from August 2023 - January 2025. We studied six species from five non-passerine orders: Cariamiformes (*Cariama cristata*), Falconiformes (*Caracara plancus* and *Daptrius chimachima*), Galliformes (*Penelope obscura*), Gruiformes (*Aramides cajaneus*), and Strigiformes (*Megascops choliba*). These birds arrived at the center after being either discovered with significant injuries (as above; **Figure 1**) or found in homes within urban or suburban environments. In the latter scenario, residents often reach out to the city’s environmental services, a program designed to rescue wildlife in residential areas. All individuals originated from Minas Gerais state, primarily from the Belo Horizonte metropolitan area and nearby cities. Upon arrival at the center, veterinarians conducted thorough evaluations of each individual, assessing body condition based on keel prominence (poor condition = pronounced keel; good condition = normal keel). Bird intake into the facility also included: (a) checking for visual signs of trichomoniasis, such as oral plaques, throat lesions, or difficulty swallowing (Dudek et al., 2018; Alrefaei et al., 2019), (b) inspecting all plumage and bare parts for the presence of ectoparasites; and the presence of any major injuries, including bone displacements, fractures, skin or eye wounds, leg injuries, and burns. Additionally, the veterinarians categorized the age of each bird (as either juvenile or adult, based on plumage characteristics and skull ossification; Von-Matter et al., 2010) and collected blood samples from the brachial vein. Unfortunately, sex of each bird was not always evident or determined, but this is unlikely to significantly impact our findings, as most of the species examined exhibit minimal or no sexual dimorphism. The samples were used to prepare blood smear slides and fill microhematocrit capillaries, and the remainder was stored in microtubes with 70% ethanol for further molecular analysis. The smears were then screened for haemosporidian parasites and H/L ratios (see more below).

### Hematological parameters

Microhematocrit evaluation involved measuring the proportion of red blood cells (RBCs) in a blood sample, which is a useful indicator of an individual’s overall health and oxygen-carrying capacity (Clark et al., 2009). To perform this procedure, a small volume of blood was drawn into a capillary tube, which was then sealed at one end with clay. The tube was placed in a centrifuge and spun at 10000 G for 3 minutes, to separate the blood into its components. Following centrifugation, blood separates into distinct layers according to density: erythrocytes form a dense layer at the bottom, plasma forms the upper layer, and a thin intermediate layer appears between them, composed mainly of leukocytes and thrombocytes. The length of the RBC column was then measured using a microhematocrit card reader and was expressed as a percentage of the total blood column length (Mondal and Zubair, 2025), which can indicate conditions such as anemia, dehydration, or polycythemia. To analyze H/L ratios, a blood smear was prepared by spreading a drop of blood thinly across a microscope slide. The smear was then air-dried, fixed with 100% methanol for 3 minutes, and stained using a 10% Giemsa solution for 70 minutes (Valkiunas, 2005). The stained slides were examined under an Olympus CX31 light microscope at 1000× magnification with oil immersion to identify heterophils (characterized by their lobed nuclei and granular cytoplasm) and lymphocytes (smaller cells with a large, round nucleus and scant cytoplasm). 22,000 total cells were counted per slide (i.e. per animal) to calculate the relative proportions of each type. The H/L ratio was then determined by dividing the number of heterophils by the number of lymphocytes. An elevated H/L ratio may indicate chronic stress, infection, or inflammation, whereas a lower ratio can reflect baseline immune conditions (Satyaningtijas et al., 2020).

### Microscopy and blood parasite identification and quantification

The stained slides were also used to identify parasite forms under an Olympus CX31 light microscope at 1000× magnification with oil immersion. Haemosporidian parasite identification (*Plasmodium* spp. and *Haemoproteus* spp.) followed established and well-described protocols (Valkiunas, 2005). Infection severity was then quantified by analyzing 200 microscopic fields with monolayered blood cells, counting parasitized cells among 22,000 total cells.

### DNA extraction and cytochrome b gene amplification

To ensure the detection of *Haemoproteus* spp. and *Plasmodium* spp. infections in cases where parasitemia was low and difficult to identify through microscopy, we also performed PCR screening for haemosporidian parasites. For DNA extraction, a 20 μL volume of blood was placed in 1.5 mL microtubes and incubated at 60°C until the alcohol was completely evaporated. Then, 100 μL of 5% Chelex 100 (Sigma-Aldrich, Saint Louis, Missouri, USA) and 2.75 μL of proteinase K (20 mg/mL) (Phoneutria, Minas Gerais, Brazil) were added to the samples. The tubes were briefly vortexed and incubated at 60°C for 2 hours. After digestion, an enzymatic denaturation was performed at 95°C for 10 minutes. The tubes were then cooled to room temperature, vortexed, and centrifuged at 11,000 rpm for 10 minutes. The supernatant containing DNA was collected and quantified using a NanoDrop 2000 spectrophotometer (Thermo Scientific, USA) to ensure DNA concentrations were within the optimal range (40–80 ng/µL) (Pacheco et al., 2018) for further analysis and stored at –20°C. Positive control samples (*Plasmodium falciparum*) and negative control samples (MilliQ water) were also subjected to the extraction process. A 1 µL aliquot of the extracted DNA was subjected to a nested PCR protocol targeting a 478 bp region of the mitochondrial Cyt b gene of *Plasmodium* spp., and *Haemoproteus* spp. species (Hellgren et al., 2004), using the recently extracted positive and negative controls, as well as specific controls to ensure the reaction’s functionality (*P. falciparum*, which was previously amplified in other reactions, and MilliQ water). PCR products were visualized on 6% polyacrylamide gels stained with silver nitrate to confirm amplification. An individual was classified as infected if PCR and/or microscopy yielded a positive result.

### Statistical analysis

#### Haemosporidian parasite occurrence

All analyses were performed with R software (R Core Team, 2024). We visually assessed the two numerical variables, microhematocrit levels and H/L ratios, for homoscedasticity using histograms. H/L ratios showed a right-skewed distribution, which we corrected using a square-root transformation. We scaled microhematocrit levels and H/L ratios to have a mean of zero and standard deviation of one using the *scale* function (R Core Team, 2024) to make all numeric variables on similar scales. To account for potential temporal variations in infection likelihood, we divided the dataset into dry (April to September) and wet (October to March) seasons.

We used three measurements of malaria occurrence in our analyses: presence/absence of (1) only *Plasmodium* spp., (2) only *Haemoproteus* spp., and (3) either genus (i.e. combined parasite occurrence). Thus, we constructed three separate generalized linear mixed model (GLMM) using the *glmer* function from the *lme4* package (Bates et al., 2015) to examine predictors of these three parasite-occurrence variables. For the *Plasmodium* spp. and *Haemoproteus* spp. specific models, total sample size was slightly smaller (*n* = 71 in each, compared to *n* = 75 for the combined parasite occurrence model) because we could not obtain clear PCR sequencing results for some samples, only confirming a positive infection status without genus-level identification. Each model included the following predictors: microhematocrit level, H/L ratio, occurrence of major injuries, age class, arrival body condition, tick occurrence, trichomoniasis occurrence, and season (dry vs. wet). We also included species identity as a random factor to account for both phylogenetic relatedness and interspecific variation. We tested for the absence of multicollinearity among predictors using the variance inflation factor (VIF), calculated with the *VIF* function from the *regclass* package (Petrie, 2020). A threshold of two for was used to identify collinear predictors, which made us exclude tick occurrence from all models. To determine the importance of explanatory variables, we employed an information-theoretic approach (Burnham and Anderson, 2002). Using the *dredge* function from the *MuMIn* package (Barton, 2019), we generated all possible models with the selected explanatory variables. For cases where the best model had a weight of less than 0.8, we calculated model-averaged estimates using the *model.avg* function from the same package. If the best model had a weight greater than 0.8, we focused exclusively on analyzing that model. Finally, we selected the most important explanatory variables based on their estimate, conditional standard error, and 95% confidence interval. We evaluated model performance using root mean square error (RMSE), with lower RMSE values indicating better model fit. Therefore, models with RMSE values closer to zero were considered to provide a good fit to the data (Norberg et al., 2019; Tobler et al., 2019).

We also evaluated predictors of hemoparasite severity – defined as the proportion of infected versus uninfected red blood cells (combining *Plasmodium* spp. and *Haemoproteus* spp.) – in the 28 malaria-infected birds. Due to the left-skewed distribution of severity values, we log-transformed both malaria severity and H/L ratio for analysis. Following this, we employed a similar modeling approach to that used for the occurrence model, focusing on model selection and identifying statistically significant predictors. We did not identify any predictors with a high Variance Inflation Factor (VIF), so all predictors were retained in the model, including ectoparasite occurrence.

## Results

**Figure 2:**
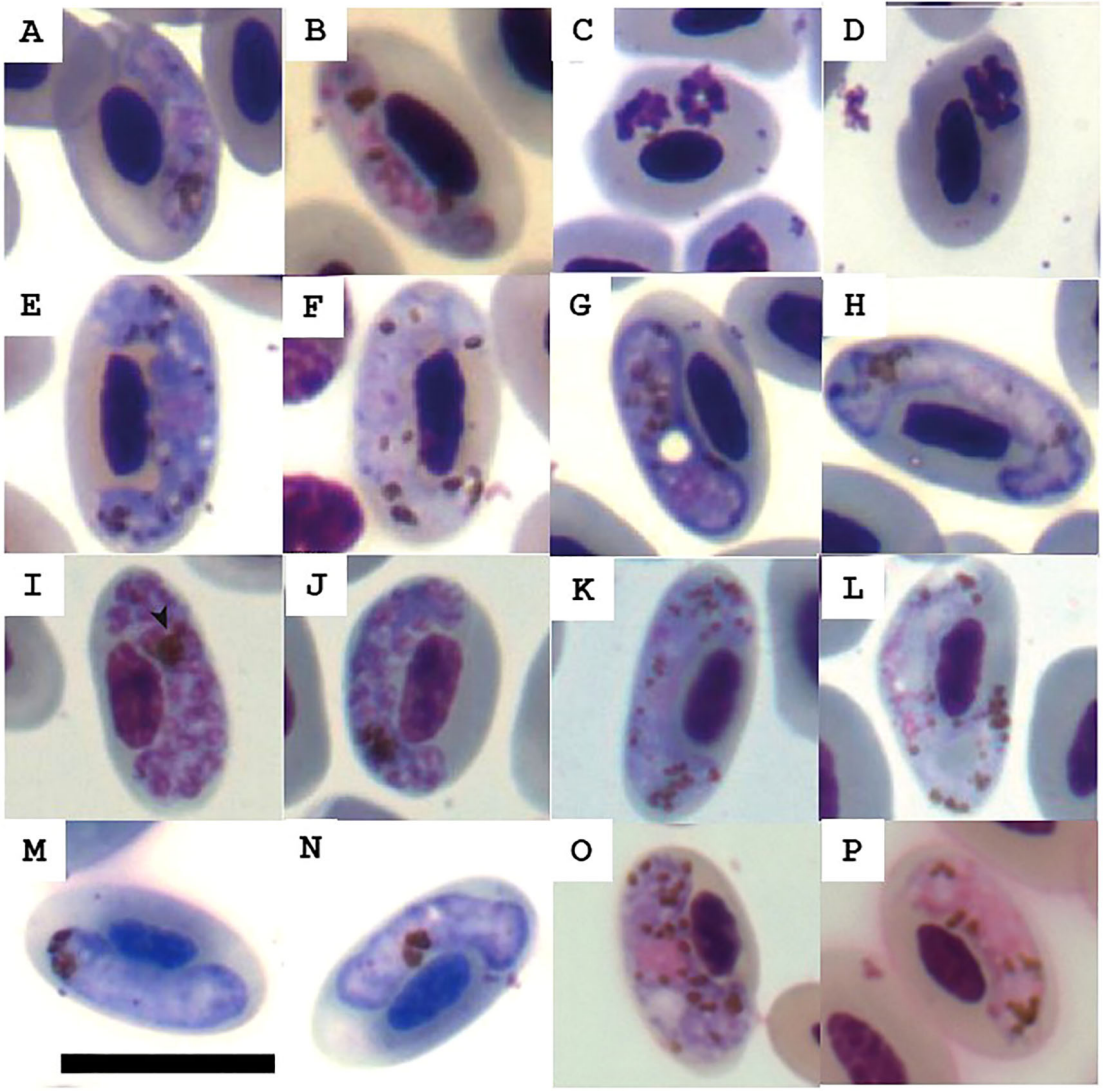
Haemosporidian morphotypes found in peripheral blood of avian hosts. **(A-D)** *Plasmodium* sp. from *Aramides cajaneus*; **(E, F)** *Haemoproteus* sp. from raptors (*Caracara plancus* and *Daptrius chimachima*); **(G, H)** *Haemoproteus* sp. from *Penelope obscura*; **(I–L)** *Plasmodium* sp. from *Cariama cristata* and *D. chimachima*; **(M, N)** *Haemoproteus* sp. from *Megascops choliba*; **(O, P)** *Haemoproteus* sp. from *C. cristata*. Scale bar = 10 mm.

Across all sampled species, the average proportion of individuals infected with malarial parasites was 37%, with *Aramides cajaneus* exhibiting the highest prevalence (71%). Mean malaria severity was 0.002 ± 0.002, with *Caracara plancus* showing the highest value (0.006) (**Table 1**; **Figure 2**). Trichomoniasis prevalence was generally low (9%), with *Daptrius chimachima* having the highest occurrence (40%). Additionally, ectoparasitic were found in only 7% of individuals, specifically in four *Caracara plancus* and one *Cariama cristata*. Injuries were observed in 37% of individuals, with *Cariama cristata* showing the highest proportion (61.5%). Lastly, mean microhematocrit and H/L ratio were 0.45 ± 0.10 and 4.0 ± 5.2, respectively, with *Daptrius chimachima* having the highest H/L ratio (8.4 ± 13.4) and *Penelope obscura* the highest microhematocrit (0.50 ± 0.07).

**Table 1:** Data summary for the study species, alongside their taxonomic classification.

Order	Family	Species	#	I	M	T	S	H	HL
Cariamiformes	Cariamidae	*Cariama cristata*	13	8	9	0	0.001 ± 0.002	0.41 ± 0.11	5.7 ± 3.5
Falconiformes	Falconidae	*Caracara plancus*	28	8	3	5	0.006 ± 0.011	0.47 ± 0.12	3.8 ± 4.4
		*Daptrius chimachima*	5	3	2	2	0.001 ± 0.001	0.45 ± 0.04	8.4 ± 13.4
Galliformes	Cracidae	*Penelope obscura*	5	2	3	0	0.000 ± 0.000	0.50 ± 0.07	4.1 ± 4.6
Gruiformes	Rallidae	*Aramides cajaneus*	7	4	5	0	0.001 ± 0.002	0.46 ± 0.09	1.8 ± 2.7
Strigiformes	Strigidae	*Megascops choliba*	17	3	6	0	0.003 ± 0.006	0.44 ± 0.02	2.6 ± 4.5
Total			75	28	28	7	0.002 ± 0004	0.45 ± 0.10	4.0 ± 5.2

Below we indicate number of individuals sampled per species (#), number of injured individuals (I), number of individuals infected with malarial parasites (M), number of individuals with trichomoniasis (T), mean overall haemosporidian parasite severity (E), mean microhematocrit (H), and mean H/L ratio.

**Figure 3:**
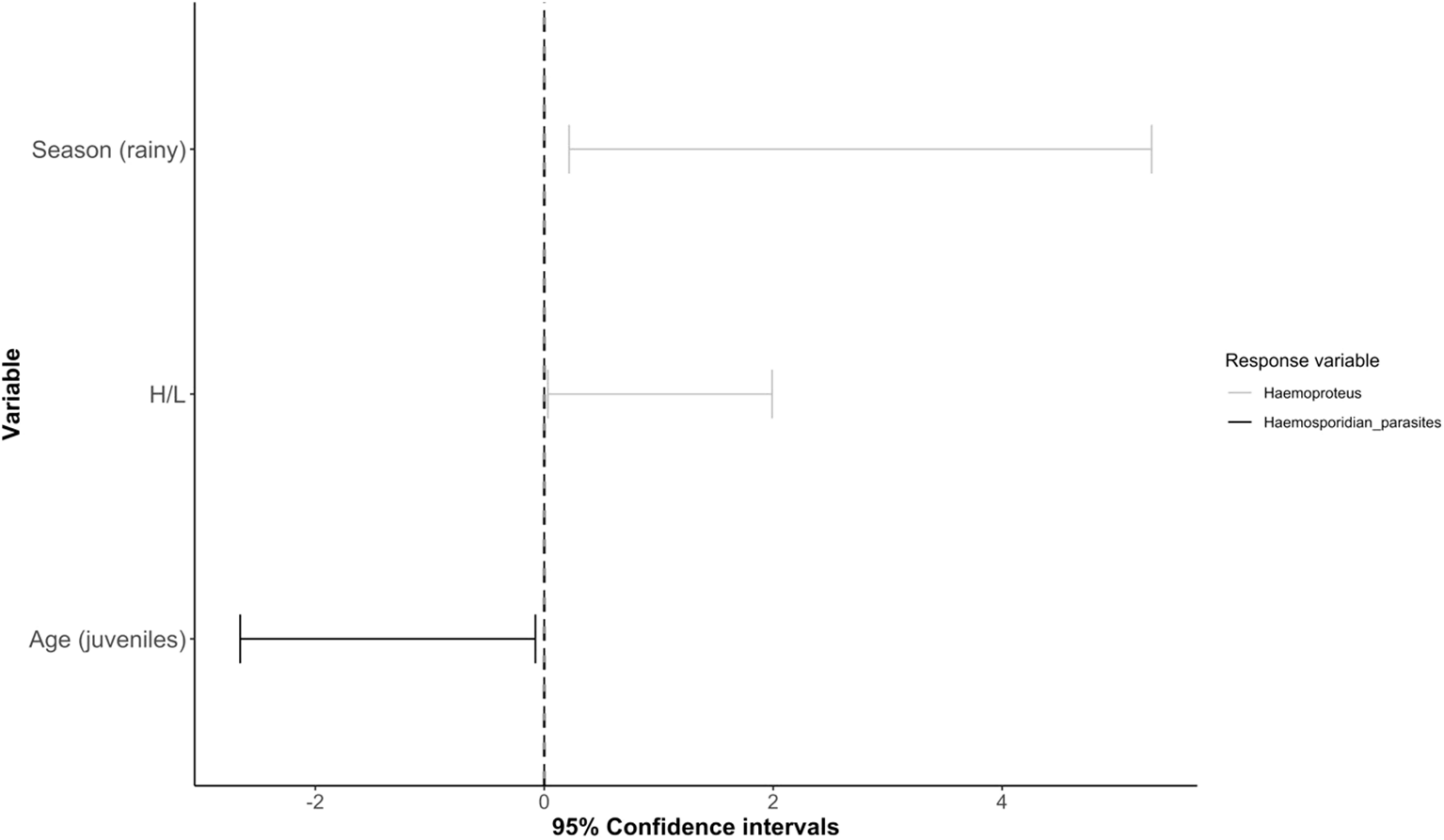
Forest plot showing the generalized linear mixed models (GLMM) 95% confidence intervals for models predicting the occurrence of haemosporidian parasites (*Plasmodium* spp. and *Haemoproteus* spp. together) and *Haemoproteus* spp. in non-passerine birds. Significant predictors are shown on the y-axis; the responses are represented by separate lines, with bars indicating confidence intervals (*Haemoproteus* spp. occurrence in gray and haemosporidian parasites in black). For complete list of predictors, please refer to **Table 2**.

The best model predicting overall haemosporidian parasite occurrence (*Plasmodium* spp. and *Haemoproteus* spp. combined; RMSE = 0.96) included only age (**Supplementary Table 1**), with juveniles being less likely than adults to be infected (**Table 2**; **Figure 3**). For *Haemoproteus* spp. (RMSE = 0.67), the best model included age, H/L ratio, and season, though only H/L ratio and season were significant predictors (**Table 2**). Individuals captured during the rainy season and those with higher H/L ratios were more likely to be infected with *Haemoproteus* spp. (**Table 2**; **Figure 3**). We did not identify any significant predictors for *Plasmodium* spp. occurrence (RMSE = 0.75) or for severity of malaria infection (RMSE = 1.65; **Supplementary Table 1**; **Table 2**).

**Table 2:** Summary of the generalized linear mixed models (GLMM) results for the occurrence of haemosporidian parasites (*Plasmodium* spp. and *Haemoproteus* spp. occurrence together), *Plasmodium* spp., and *Haemoproteus* spp., as well as haemosporidian severity (*Plasmodium* spp. and *Haemoproteus* spp. combined) in non-passerine birds.

Variable	Estimate	S.E.	95% C.I.
Response variable: haemosporidian parasite occurrence (*Plasmodium* spp. and *Haemoproteus* spp. together)			
Intercept^A^	0.06	0.65	-1.23; 1.37
**Age**	**-1.36**	**0.64**	**-2.65; -0.07**
Condition	-0.89	0.71	-2.32; 0.53
Microhematocrit level	0.27	0.32	-0.37; 0.91
*Trichomonas* spp. occurrence	0.85	1.21	-1.56; 3.27
Season	0.88	0.68	-0.48; 2.24
H/L ratio	0.25	0.31	-0.36; 0.88
Major injuries	0.37	0.68	-0.99; 1.73
Response variable: *Plasmodium* spp. occurrence			
**Intercept^B^**	**-1.73**	**0.82**	**-3.37; -0.08**
Age	-0.83	0.99	-2.82; 1.16
Condition	-0.72	1.02	-2.77; 1.32
Microhematocrit level	0.21	0.47	-0.72; 1.15
*Trichomonas* spp. occurrence	0.87	1.44	-2.00; 3.75
Season	-1.32	1.11	-3.54; 0.90
H/L ratio	-0.33	0.49	-1.31; 0.64
Major injuries	0.41	0.92	-1.42; 2.25
Response variable: *Haemoproteus* spp. occurrence			
**Intercept^C^**	**-2.38**	**1.07**	**-4.52; -0.24**
Age	-2.27	1.18	-4.63; 0.09
Condition	-0.23	1.09	-2.42; 1;95
Microhematocrit level	0.46	0.49	-0.52; 1;45
*Trichomonas* spp. occurrence	1.70	2.14	-2.57; 5.91
**Season**	**2.76**	**1.18**	**0.21; 5.30**
**H/L ratio**	**1.01**	**0.49**	**0.03; 1.99**
Major injuries	-0.08	0.96	-2.01; 1.83
Response variable: Haemosporidian parasite parasitemia			
**Intercept^D^**	**-7.30**	**0.63**	**-8.59; -6.01**
Tick occurrence	1.62	1.47	-4.68; 4.68
Age	-1.20	0.84	-2.95; 0.54
Condition	0.42	0.98	-1.60; 2.45
Microhematocrit levels	-0.21	0.40	-1.06; 0.62
*Trichomonas* spp. occurrence	-0.04	1.49	-3.14; 3.05
Season	0.70	0.76	-0.89; 2.29
H/L ratio	0.09	0.41	-0.76; 0.95
Major injuries	-0.96	0.84	-2.72; 0.78

^A^ Random factor: host binomial species: variance = 0.90; standard deviation = 0.95; ^B^ Random factor: host binomial species: variance = 1.19; standard deviation = 1.09; ^C^ Random factor: host binomial species: variance = 2.64; standard deviation = 1.62; ^D^ Random factor: host binomial species: variance = 4.03; standard deviation = 2.00.

The table includes estimated coefficients, standard error (S.E.), 95% confidence intervals (95% C.I.), and associated predictors. Significant predictors (95% C.I. without overlapping zero) are indicated in bold. Estimates reflect the effect sizes for each variable on the response variables.

The bold values represent statistically significant predictors of haemosporidian parasite occurrence. These are variables for which the 95% confidence interval (C.I.) does not include zero, indicating a significant association with the response variable at the 0.05 level.

To assess whether uneven species representation influenced our results, we re-ran all models using only the four most represented species (*Caracara plancus*, *Megascops choliba*, *Cariama cristata*, and *Aramides cajanea*), each with more than five individuals in the dataset. The results were highly consistent with those from the full dataset: in the general haemosporidian model, age remained marginally significant, and in the *Haemoproteus*-specific model, season was again marginally associated with infection risk. These findings suggest that the main patterns are not driven solely by uneven sampling across species. Additionally, we examined the distribution of individuals across species, age classes, and seasons (**Supplementary Table S4**). Notably, species with high infection prevalence—such as *Aramides cajanea* and *Cariama cristata*—were not disproportionately represented in the rainy season or in a particular age group. For example, *Aramides cajanea* was represented only by adults and almost evenly between dry (n = 4) and rainy (n = 3) seasons. These results indicate that the observed effects of age and season are not artifacts of biased sampling but instead reflect broader trends in our data.

## Discussion

We found that the occurrence of haemosporidian parasite infections (*Plasmodium* spp. and *Haemoproteus* spp. combined) in non-passerine birds was influenced by the age of the avian hosts, with juvenile birds less likely to be infected than adults. We also observed that individuals infected specifically with *Haemoproteus* spp. had a higher H/L ratio and were more often brought into the facility during the rainy season. Taken together, these findings highlight the complex interplay between host age, physiological condition, and environmental factors in shaping the dynamics of malaria parasite infections, offering new insights into the eco-immunological and life-history drivers of hemoparasite infection in tropical non-passerine birds.

First, we found that juvenile birds exhibited a lower overall occurrence of avian malaria (*Plasmodium* spp. and *Haemoproteus* spp. combined) compared to adults, contrary to our initial prediction. We propose two non-mutually exclusive explanations for this pattern: (a) chronic infections may accumulate as birds age, which have been demonstrated in several bird species, such as in white-banded tanagers (*Neothraupis fasciata*; Fecchio et al., 2015), house martins (Marzal et al., 2016), dark-eyed juncos (*Junco hyemalis*; Slowinski et al., 2022), European bee-eaters (*Merops apiaster*; Emmenegger et al., 2020), and in a community-level avian study (Ellis et al., 2014), or (b) Infections may disproportionately impact the survival of younger birds (e.g. due to their developing immune systems; Ricklefs, 1992), leading to their reduced representation in the population. For instance, in yellowthroats (*Geothlypis trichas*), older males had a higher likelihood of surviving and managing chronic haemosporidian infections compared to their younger counterparts (Freeman-Gallant and Taff, 2017). Additionally, mortality linked to haemosporidian parasites has been documented in younger cranes (from 10 different species) at a zoo in China, further highlighting the vulnerability of younger individuals to these infections (Jia et al., 2018). These findings underscore the need for further research to disentangle the mechanisms driving age-related patterns of haemosporidian parasite occurrence in passerine and non-passerine bird populations.

Second, as we predicted, individuals infected with *Haemoproteus* spp. exhibited significantly higher H/L ratios compared to uninfected individuals. We hypothesize that *Haemoproteus* spp. infections may deplete B-cells, which are responsible for humoral immunity, leading to reduced lymphocyte levels in infected individuals (Delhaye et al., 2018). Similar patterns have been observed across various passerine and non-passerine species, including reed warblers (*Acrocephalus scirpaceus*) and sedge warblers (*Acrocephalus schoenobaenus*) infected with multiple blood parasites (Wojczulanis-Jakubas et al., 2012), various owl species (Martín-Maldonado et al., 2023), and several Neotropical birds such as ruddy ground-dove (*Columbina talpacoti*), great kiskadee (*Pitangus sulphuratus*), lesser elaenia (*Elaenia chiriquensis*), flavescent warblers (*Myiothlypis flaveola*), and brown-crested flycatchers (*Myiarchus tyrannulus*; da Silva Rodrigues et al., 2021), as well as house sparrows (*Passer domesticus*; Santiago-Alarcon et al., 2018) and house finches (*Haemorhous mexicanus*; Aguiar De Souza Penha et al., 2024). However, some studies have reported no such associations in red crossbills (*Loxia curvirostra*; Cornelius et al., 2014), American redstarts (*Setophaga ruticilla*), gray catbirds (*Dumetella carolinensis*), cedar waxwings (*Bombycilla cedrorum*), red-eyed vireos (*Vireo olivaceus*; Granthon and Williams, 2017), and rosy starlings (*Pastor roseus*; Dimitrov et al., 2019), indicating that this relationship can vary across species and contexts. In addition, also consistent with our predictions, we found that *Haemoproteus* spp. infections occurred more frequently in birds brought in during the rainy season. This result suggests that the higher prevalence of *Haemoproteus* spp. during the rainy season may overlap with optimal conditions for vector development and proliferation, given the more humid and wetter conditions (Meneghim et al., 2021). The tropical-bird literature offers mixed support for this pattern, with some studies reporting a higher malaria prevalence at the end of the dry season (Ferreira et al., 2017; Pulgarin et al., 2018; de Angeli Dutra et al., 2023), others finding no seasonal trends (Fecchio et al., 2015), and yet others reporting higher occurrence during the wet season (Schultz et al., 2011; Rivero de Aguilar et al., 2018). These findings illustrate the importance of considering environmental factors like climate and water availability when studying host-parasite interactions and emphasize the complexity of *Haemoproteus* spp. epidemiology.

We did not find any evidence that body condition, trichomoniasis, tick presence, or major injuries were statistically significant predictors of haemosporidian parasite occurrence, either overall (*Plasmodium* spp. and *Haemoproteus* spp. combined) or when analyzed separately. These results suggest that poor physical condition in affected individuals may be attributed to other causes, such as accidental collisions or encounters with predators (Stenkat et al., 2013), rather than parasitic infection. The lack of association with *Trichomonas* spp. or ectoparasites also underscores the variability in susceptibility to co-infections, which may be shaped by species-specific differences in ecology, physiology, and immune function. Associations in other bird species between haemosporidian parasites and pathogens such as *Mycoplasma gallisepticum* (Reinoso-Perez et al., 2020), *Trypanosoma* spp (Soares et al., 2016a), *Isospora* sp (Aguiar De Souza Penha et al., 2024), microfilariae (Clark et al., 2016), and *Trichomonas gallinae* (Thomas et al., 2022) demonstrate the complexity of host-pathogen interactions. This variability makes it challenging to establish consistent patterns of co-infection and emphasizes the need for targeted experimental studies to better understand the mechanisms driving these interactions and the broader ecological and evolutionary dynamics of co-infections across clades and geographic regions. It is also noteworthy that the lack of significant patterns in *Trichomonas* spp. and tick infections may be attributable to the low prevalence of these parasites in our sample (9% and 7%, respectively). Interestingly, microhematocrit levels were also not an important predictor of any of our haemosporidian parasite measurements (occurrence and severity), despite the fact that, based on other studies, we expected it would be reduced in infected individuals (Ellis et al., 2014; but see Martín-Maldonado et al., 2023).

Lastly, we found no significant predictors of hemoparasite severity in the 28 malaria-infected birds in this study. This contrasts with findings from studies of other non-passerine and/or tropical species, which have identified various factors influencing malaria severity, such as body condition, H/L ratio, total white-blood cell count, and reproductive effort (Knowles et al., 2009; da Silva Rodrigues et al., 2021). These results suggest that the drivers of hemoparasite severity may be context-dependent and demonstrate the need for further research with larger sample sizes and broader ecological contexts to better understand the patterns and determinants of avian malaria severity.

We must also address the fact that our study was conducted on a sample of rescued and rehabilitated birds, many of which were injured or otherwise compromised, and not of free-ranging, wholly uninjured individuals. As a result, our findings should be interpreted cautiously, as health/stress conditions related to injury or a home invasion could have influenced parasitological and physiological measures. Sampling free-ranging, uninjured birds in the wild would provide a valuable comparison to evaluate whether the patterns observed in these rescued individuals hold true in natural populations. Additionally, lack of data on the sex of the birds was a limitation, as we were unable to account for potential sex differences in infection or physiology; we recommend that future studies of this nature incorporate sex of the animals.

In summary, our study provides novel insight into the eco-immunological correlates of haemosporidian infections in non-passerine birds, a group that remains largely underrepresented in avian malaria research. Although the sample size was limited, our results consistently point to age and seasonality—particularly in *Haemoproteus* spp. infections—as key drivers of infection patterns, with additional support for the role of host stress physiology (via H/L ratio). These patterns were robust even when accounting for interspecific differences and restricting analyses to the most represented species. While not all variables yielded significant associations, our findings underscore the multifactorial nature of infection risk and highlight the importance of integrating life-history, physiological, and environmental factors to understand host–parasite dynamics in tropical bird assemblages. Further research with larger and free-ranging populations will be essential to refine these associations and explore their broader conservation and epidemiological implications.

## Data Availability

The original contributions presented in the study are publicly available. The data presented in the study are deposited in the GitHub repository, accessible at https://github.com/victoraspenha/FrontPara_Lis-et-al-2025-.

## References

[B1] Aguiar De Souza Penha V. Manica L. T. Barrand Z. A. Hepp C. M. McGraw K. J. (2024). Correlates of co-infection with coccidiosis and avian malaria in house finches (*Haemorhous mexicanus*). J. Wildl. Dis. 61, 1–13. doi: 10.7589/JWD-D-23-00175, PMID: 38741368

[B2] Alrefaei A. F. Low R. Hall N. Jardim R. Dávila A. Gerhold R.. (2019). Multilocus analysis resolves the european finch epidemic strain of *trichomonas gallinae* and suggests introgression from divergent trichomonads. Genome Biol. Evol. 11, 2391–2402. doi: 10.1093/gbe/evz164, PMID: 31364699 PMC6735722

[B3] Barton K. (2019). *MuMIn: Multi-model inference* (R package version 1.43.15) [Computer software]. (Vienna, Austria: R Foundation for Statistical Computing). Available online at: https://CRAN.R-project.org/package=MuMIn (Accessed January 18, 2025).

[B4] Bates D. Maechler M. Bolker B. Walker S. (2015). Fitting linear mixed-effects models using lme4. J. Stat. Softw 67, 1–48. doi: 10.18637/jss.v067.i01

[B5] Bell J. A. González-Acuña D. Tkach Vasyl V. (2020). Haemosporidian parasites of Chilean ducks: the importance of biogeography and nonpasserine hosts. J. Parasitol. 106, 211. doi: 10.1645/19-130, PMID: 32164026

[B6] Burnham K. P. Anderson D. R. (2002). Model selection and inference: A practical information-theoretic approach. 2nd edn (New York: Springer).

[B7] Capasso S. Schumm Y. R. Quillfeldt P. Bonsergent C. Malandrin L. Lorenti E.. (2023). Surveillance of avian malaria and related haemoparasites in common terns (*Sterna hirundo*) on the Atlantic coast of South America. Parasitology 150, 498–504. doi: 10.1017/S0031182023000185, PMID: 36892015 PMC10260293

[B8] Capua I. Mutinelli F. Bozza M. A. Terregino C. Cattoli G. (2000). Highly pathogenic avian influenza (H7N1) in ostriches (*Struthio camelus*). Avian Pathol. 29, 643–646. doi: 10.1080/03079450020016913, PMID: 19184863

[B9] Carlson J. S. Walther E. Trout Fryxell R. Staley S. Tell L. A. Sehgal R. N. M.. (2015). Identifying avian malaria vectors: Sampling methods influence outcomes. Parasit Vectors 8, 365. doi: 10.1186/s13071-015-0969-0, PMID: 26160384 PMC4702297

[B10] Clark P. Boardman W. Raidal S. (2009). Atlas of clinical avian hematology (Hoboken, New Jersey: Wiley-Blackwell).

[B11] Clark N. J. Wells K. Dimitrov D. Clegg S. M. (2016). Co-infections and environmental conditions drive the distributions of blood parasites in wild birds. J. Anim. Ecol. 85, 1461–1470. doi: 10.1111/1365-2656.12578, PMID: 27561363

[B12] Colombo A. F. Joly C. A. (2010). Brazilian Atlantic Forest lato sensu: the most ancient Brazilian forest, and a biodiversity hotspot, is highly threatened by climate change. Braz. J. Biol. 70, 697–708. doi: 10.1590/S1519-69842010000400002, PMID: 21085776

[B13] Cornelius E. A. Davis A. K. Altizer S. A. (2014). How important are hemoparasites to migratory songbirds? Evaluating physiological measures and infection status in three neotropical migrants during stopover. Physiol. Biochem. Zoology 87, 719–728. doi: 10.1086/677541, PMID: 25244383

[B14] da Silva Rodrigues R. de Souza Penha V. A. Miwa R. Y. Branco J. O. Junior O. M. (2021). Stress and body condition predict haemosporidian parasitaemia in birds from cerrado, southeastern Brazil. Ardea 109, 175–183. doi: 10.5253/arde.v109i3.a7

[B15] de Angeli Dutra D. Khan A. U. Ferreira F. C. Beirão M. V. Pichorim M. Moreira P. A.. (2023). Host phylogeny and seasonality shapes avian haemosporidian prevalence in a Brazilian biodiverse and dry forest: the Caatinga. Parasitology 150, 1277–1285. doi: 10.1017/S0031182023000549, PMID: 37246557 PMC10941212

[B16] Delhaye J. Jenkins T. Glaizot O. Christe P. (2018). Avian malaria and bird humoral immune response. Malar J. 17, 1–7. doi: 10.1186/s12936-018-2219-3, PMID: 29426311 PMC5807826

[B17] Dimitrov D. Marinov M. P. Bobeva A. Ilieva M. Bedev K. Atanasov T.. (2019). Haemosporidian parasites and leukocyte profiles of pre-migratory rosy starlings (*Pastor roseus*) brought into captivity. Anim. Migration 6, 41–48. doi: 10.1515/ami-2019-0005

[B18] Dinhopl N. Nedorost N. Mostegl M. M. Weissenbacher-Lang C. Weissenböck H. (2015). *In situ* hybridization and sequence analysis reveal an association of *Plasmodium* spp. with mortalities in wild passerine birds in Austria. Parasitol. Res. 114, 1455–1462. doi: 10.1007/s00436-015-4328-z, PMID: 25636246

[B19] Dudek B. M. Kochert M. N. Barnes J. G. Bloom P. H. Papp J. M. Gerhold R. W.. (2018). Prevalence and risk factors of *Trichomonas gallinae* and trichomoniasis in golden eagle (*Aquila chrysaetos*) nestlings in western North America. J. Wildl Dis. 54, 755–764. doi: 10.7589/2017-11-271, PMID: 29863970

[B20] Ellis V. A. Kunkel M. R. Ricklefs R. E. (2014). The ecology of host immune responses to chronic avian haemosporidian infection. Oecologia 176, 729–737. doi: 10.1007/s00442-014-3048-x, PMID: 25179282

[B21] Emmenegger T. Alves J. A. Rocha A. D. Costa J. S. Schmid R. Schulze M.. (2020). Population- and age-specific patterns of haemosporidian assemblages and infection levels in European bee-eaters (*Merops apiaster*). Int. J. Parasitol. 50, 1125–1131. doi: 10.1016/j.ijpara.2020.07.005, PMID: 32866492

[B22] Ezenwa V. O. Godsey M. S. King R. J. Guptill S. C. (2006). Avian diversity and West Nile virus: testing associations between biodiversity and infectious disease risk. Proc. R. Soc. B: Biol. Sci. 273, 109–117. doi: 10.1098/rspb.2005.3284, PMID: 16519242 PMC1560012

[B23] Fecchio A. Clark N. J. Bell J. A. Skeen H. R. Lutz H. L. De La Torre G. M.. (2021a). Global drivers of avian haemosporidian infections vary across zoogeographical regions. Global Ecol. Biogeography 30, 2393–2406. doi: 10.1111/geb.13390

[B24] Fecchio A. Dias R. I. Ferreira T. V. Reyes A. O. Dispoto J. H. Weckstein J. D.. (2022). Host foraging behavior and nest type influence prevalence of avian haemosporidian parasites in the Pantanal. Parasitol. Res. 121, 1407–1417. doi: 10.1007/s00436-022-07453-3, PMID: 35106653

[B25] Fecchio A. Lima M. R. Bell J. A. Schunck F. Corrêa A. H. Beco R.. (2021b). Loss of forest cover and host functional diversity increases prevalence of avian malaria parasites in the Atlantic Forest. Int. J. Parasitol. 51, 719–728. doi: 10.1016/j.ijpara.2021.01.001, PMID: 33722680

[B26] Fecchio A. Lima M. R. Silveira P. Ribas A. C. A. Caparroz R. Marini M. Â. (2015). Age, but not sex and seasonality, influence Haemosporida prevalence in White-banded Tanagers (*Neothraupis fasciata*) from central Brazil. Can. J. Zool 93, 71–77. doi: 10.1139/cjz-2014-0119

[B27] Fecchio A. Ribeiro R. M. Ferreira F. C. de Angeli Dutra D. Tolesano-Pascoli G. Alquezar R. D.. (2021c). Higher infection probability of haemosporidian parasites in Blue-black Grassquits (*Volatinia jacarina*) inhabiting native vegetation across Brazil. Parasitol. Int. 80, 102204. doi: 10.1016/j.parint.2020.102204, PMID: 33045411

[B28] Fenton H. McManamon R. Howerth E. W. (2018). “Anseriformes, ciconiiformes, charadriiformes, and gruiformes,” in Pathology of wildlife and zoo animals (London, United Kingdom: Elsevier), 697–721. doi: 10.1016/B978-0-12-805306-5.00029-8

[B29] Ferreira F. C. Rodrigues R. A. Ellis V. A. Leite L. O. Borges M. A. Z. Braga E. M. (2017). Habitat modification and seasonality influence avian haemosporidian parasite distributions in southeastern Brazil. PloS One 12, 1–18. doi: 10.1371/journal.pone.0178791, PMID: 28575046 PMC5456369

[B30] Ferreira-Junior F. C. de Angeli Dutra D. Silveira P. Pacheco R. C. Witter R. de Souza Ramos D. G.. (2018). A new pathogen spillover from domestic to wild animals: *Plasmodium juxtanucleare* infects free-living passerines in Brazil. Parasitology 145, 1949–1958. doi: 10.1017/S003118201800077X, PMID: 29739479

[B31] Freeman-Gallant C. R. Taff C. C. (2017). Age-specific patterns of infection with haemosporidians and trypanosomes in a warbler: implications for sexual selection. Oecologia 184, 813–823. doi: 10.1007/s00442-017-3919-z, PMID: 28756490

[B32] Gill F. (2007). Ornithology, third edit (New York: W.H. Freeman).

[B33] González-Olvera M. Hernandez-Colina A. Himmel T. Eckley L. Lopez J. Chantrey J.. (2022). Molecular and epidemiological surveillance of *Plasmodium* spp. during a mortality event affecting Humboldt penguins (*Spheniscus humboldti*) at a zoo in the UK. Int. J. Parasitol. Parasites Wildl 19, 26–37. doi: 10.1016/j.ijppaw.2022.06.010, PMID: 36035627 PMC9403903

[B34] Granthon C. Williams D. A. (2017). Avian malaria, body condition, and blood parameters in four species of songbirds. Wilson J. Ornithol 129, 492–508. doi: 10.1676/16-060.1

[B35] Haghtalab N. Moore N. Heerspink B. P. Hyndman D. W. (2020). Evaluating spatial patterns in precipitation trends across the Amazon basin driven by land cover and global scale forcings. Theor. Appl. Climatol 140, 411–427. doi: 10.1007/s00704-019-03085-3

[B36] Hellgren O. Waldenström J. Bensch S. (2004). a New Pcr Assay for Simultaneous Studies of *Leucocytozoon*, Plasmodium, and *Haemoproteus* From Avian Blood. J. Parasitol. 90, 797–802. doi: 10.1645/GE-184R1, PMID: 15357072

[B37] Hernández-Lara C. González-García F. Santiago-Alarcon D. (2017). Spatial and seasonal variation of avian malaria infections in five different land use types within a Neotropical montane forest matrix. Landsc Urban Plan 157, 151–160. doi: 10.1016/j.landurbplan.2016.05.025

[B38] Ilgūnas M. Himmel T. Harl J. Dagys M. Valkiūnas G. Weissenböck H. (2022). Exo-erythrocytic development of avian haemosporidian parasites in european owls. Animals 12, 2212. doi: 10.3390/ani12172212, PMID: 36077935 PMC9454416

[B39] Jia T. Huang X. Valkiūnas G. Yang M. Zheng C. Pu T.. (2018). Malaria parasites and related haemosporidians cause mortality in cranes: a study on the parasites diversity, prevalence and distribution in Beijing Zoo. Malar J. 17, 234. doi: 10.1186/s12936-018-2385-3, PMID: 29914492 PMC6006844

[B40] Knowles S. C. L. Nakagawa S. Sheldon B. C. (2009). Elevated reproductive effort increases blood parasitaemia and decreases immune function in birds: A meta-regression approach. Funct. Ecol. 23, 405–415. doi: 10.1111/j.1365-2435.2008.01507.x

[B41] Kocan R. M. (1968). Anemia and mechanism of erythrocyte destruction in ducks with acute *leucocytozoon* infections. J. Protozool 15, 455–462. doi: 10.1111/j.1550-7408.1968.tb02156.x, PMID: 4973746

[B42] Lotta-Arévalo I. A. González A. D. Gamboa-Suárez B. A. Pacheco M. A. Escalante A. A. Moreno C.. (2023). Haemosporidians in non-passerine birds of Colombia: an overview of the last 20 years of research. Diversity (Basel) 15, 57. doi: 10.3390/d15010057

[B43] Martín-Maldonado B. Mencía-Gutiérrez A. Andreu-Vázquez C. Fernández R. Pastor-Tiburón N. Alvarado A.. (2023). A four-year survey of hemoparasites from nocturnal raptors (Strigiformes) confirms a relation between *leucocytozoon* and low hematocrit and body condition scores of parasitized birds. Vet. Sci. 10, 54. doi: 10.3390/vetsci10010054, PMID: 36669055 PMC9865734

[B44] Marzal A. Balbontín J. Reviriego M. García-Longoria L. Relinque C. Hermosell I. G.. (2016). A longitudinal study of age-related changes in *Haemoproteus* infection in a passerine bird. Oikos 125, 1092–1099. doi: 10.1111/oik.02778

[B45] Marzal A. Reviriego M. Hermosell I. G. Balbontín J. Bensch S. Relinque C.. (2013). Malaria infection and feather growth rate predict reproductive success in house martins. Oecologia 171, 853–861. doi: 10.1007/s00442-012-2444-3, PMID: 22961369

[B46] Meneghim R. L. F. d. S. Madeira N. G. Ribolla P. E. M. Padovani C. R. Schellini S. A. (2021). Flies as possible vectors of inflammatory trachoma transmission in a Brazilian municipality. Rev. Inst Med. Trop. Sao Paulo 63, e66. doi: 10.1590/s1678-9946202163066, PMID: 34495263 PMC8428871

[B47] Mitchell E. B. Johns J. (2008). Avian hematology and related disorders. Veterinary Clinics North America: Exotic Anim. Pract. 11, 501–522. doi: 10.1016/j.cvex.2008.03.004, PMID: 18675731

[B48] Mondal H. Zubair M. (2025). “Hematocri,” in StatPearls (StatPearls Publishing, Treasure Island (FL). Available online at: https://www.ncbi.nlm.nih.gov/books/NBK542276/.

[B49] Muriel J. Marzal A. Magallanes S. García-Longoria L. Suarez-Rubio M. Bates P. J. J.. (2021). Prevalence and diversity of avian haemosporidians may vary with anthropogenic disturbance in tropical habitats in Myanmar. Diversity (Basel) 13, 111. doi: 10.3390/d13030111

[B50] Niebuhr C. N. Poulin R. Tompkins D. M. (2016). Is avian malaria playing a role in native bird declines in New Zealand? Testing hypotheses along an elevational gradient. PloS One 11, e0165918. doi: 10.1371/journal.pone.0165918, PMID: 27802326 PMC5089714

[B51] Norberg A. Abrego N. Blanchet F. G. Adler F. R. Anderson B. J. Anttila J.. (2019). A comprehensive evaluation of predictive performance of 33 species distribution models at species and community levels. Ecol. Monogr. 89, e01370. doi: 10.1002/ecm.1370

[B52] Oliveira L. Dias R. J. P. Rossi M. F. D’Agosto M. Santos H. A. (2020). Molecular diversity and coalescent species delimitation of avian haemosporidian parasites in an endemic bird species of South America. Parasitol. Res. 119, 4033–4047. doi: 10.1007/s00436-020-06908-9, PMID: 33030600

[B53] Ortiz-Catedral L. Brunton D. Stidworthy M. F. Elsheikha H. M. Pennycott T. Schulze C.. (2019). *Haemoproteus minutus* is highly virulent for Australasian and South American parrots. Parasit Vectors 12, 40. doi: 10.1186/s13071-018-3255-0, PMID: 30654841 PMC6337802

[B54] Pacheco M. A. Cepeda A. S. Bernotienė R. Lotta I. A. Matta N. E Valkiūnas G. (2018). Primers targeting mitochondrial genes of avian haemosporidians: PCR detection and differential DNA amplification of parasites belonging to different genera. Int. J. Parasitol. 48 (8), 657–670. doi: 10.1016/j.ijpara.2018.02.003, PMID: 29625126 PMC6004333

[B55] Pacheco M. A. Escalante A. A. Garner M. M. Bradley G. A. Aguilar R. F. (2011). Haemosporidian infection in captive masked bobwhite quail (*Colinus virginianus ridgwayi*), an endangered subspecies of the northern bobwhite quail. Vet. Parasitol. 182, 113–120. doi: 10.1016/j.vetpar.2011.06.006, PMID: 21726940 PMC3742108

[B56] Petrie A. (2020). “regclass: tools for an introductory class in regression and modeling,” in R package version 1.6. (Vienna, Austria: R Foundation for Statistical Computing) Available online at: https://cran.r-project.org/web/packages/regclass/. (Accessed January 18, 2025).

[B57] Poulin R. (2021). The rise of ecological parasitology: twelve landmark advances that changed its history. Int. J. Parasitol. 51, 1073–1084. doi: 10.1016/j.ijpara.2021.07.001, PMID: 34390744

[B58] Pulgarín-R P. C. Gómez J. P. Robinson S. Ricklefs R. E. Cadena C. D. (2018). Host species, and not environment, predicts variation in blood parasite prevalence, distribution, and diversity along a humidity gradient in northern South America. Ecol. Evol. 8, 3800–3814. doi: 10.1002/ece3.3785, PMID: 29721258 PMC5916302

[B59] Rahman M. M. Talukder A. Chowdhury M. M. H. Talukder R. Akter R. (2021). Coronaviruses in wild birds – A potential and suitable vector for global distribution. Vet. Med. Sci. 7, 264–272. doi: 10.1002/vms3.360, PMID: 32970935 PMC7537155

[B60] R Core Team (2024). R: A language and environment for statistical computing [Computer software]. (Vienna, Austria: R Foundation for Statistical Computing). Available online at: https://www.R-project.org/. (Accessed January 18, 2025).

[B61] Reinoso-Pérez M. T. Dhondt K. V. Sydenstricker A. V. Heylen D. Dhondt A. A. (2020). Complex interactions between bacteria and haemosporidia in coinfected hosts: An experiment. Ecol. Evol. 10, 5801–5814. doi: 10.1002/ece3.6318, PMID: 32607191 PMC7319152

[B62] Ricklefs R. E. (1992). Embryonic development period and the prevalence of avian blood parasites. Proc. Natl. Acad. Sci. U.S.A. 89, 4722–4725. doi: 10.1073/pnas.89.10.4722, PMID: 1584808 PMC49155

[B63] Ricklefs R. E. Sheldon K. S. (2007). Malaria prevalence and white-blood-cell response to infection in a tropical and in a temperate thrush. Auk 124, 1254–1266. doi: 10.1093/auk/124.4.1254

[B64] Rivero de Aguilar J. Castillo F. Moreno A. Peñafiel N. Browne L. Walter S. T.. (2018). Patterns of avian haemosporidian infections vary with time, but not habitat, in a fragmented Neotropical landscape. PloS One 13, e0206493. doi: 10.1371/journal.pone.0206493, PMID: 30379912 PMC6209335

[B65] Rodrigues R. A. Felix G. M. F. Pichorim M. Moreira P. A. Braga E. M. (2021). Host migration and environmental temperature influence avian haemosporidians prevalence: A molecular survey in a Brazilian Atlantic rainforest. PeerJ 9, e11555. doi: 10.7717/peerj.11555, PMID: 34221715 PMC8231341

[B66] Rohrer S. D. Jiménez-Uzcátegui G. Parker P. G. Chubiz L. M. (2023). Composition and function of the Galapagos penguin gut microbiome vary with age, location, and a putative bacterial pathogen. Sci. Rep. 13, 5358. doi: 10.1038/s41598-023-31826-y, PMID: 37005428 PMC10067942

[B67] Santiago-Alarcon D. Carbó-Ramírez P. Macgregor-Fors I. Chávez-Zichinelli C. A. Yeh P. J. (2018). The prevalence of avian haemosporidian parasites in an invasive bird is lower in urban than in non-urban environments. Ibis 162, 201–214. doi: 10.1111/ibi.12699

[B68] Santiago-Alarcon D. Rodríguez-Ferraro A. Parker P. G. Ricklefs R. E. (2014). Different meal, same flavor: cospeciation and host switching of haemosporidian parasites in some non-passerine birds. Parasit Vectors 7, 286. doi: 10.1186/1756-3305-7-286, PMID: 24957563 PMC4077843

[B69] Satyaningtijas A. S. Suprayogi A. Darusman H. S. Nurdin A. Hanadhita D. (2020). Relative white blood cell counts, heterophil-to-lymphocyte ratio, and discovery of blood parasites in wild dugong (*Dugong dugon*) at Lingayan Island, Toli-toli, Indonesia. Vet. World 13, 1849–1853. doi: 10.14202/vetworld.2020.1849-1853, PMID: 33132595 PMC7566243

[B70] Schultz A. Underhill L. G. Earlé R. Underhill G. (2011). Seasonality, distribution and taxonomic status of avian haemosporidian parasites within the Greater Cape Town area, South Africa. Ostrich 82, 141–153. doi: 10.2989/00306525.2011.603478

[B71] Sijbranda D. Hunter S. Howe L. Lenting B. Argilla L. Gartrell B. (2017). Cases of mortality in little penguins (*Eudyptula minor*) in New Zealand associated with avian malaria. N Z Vet. J. 65, 332–337. doi: 10.1080/00480169.2017.1359124, PMID: 28738733

[B72] Slowinski S. P. Geissler A. J. Gerlach N. Heidinger B. J. Ketterson E. D. (2022). The probability of being infected with haemosporidian parasites increases with host age but is not affected by experimental testosterone elevation in a wild songbird. J. Avian Biol. 2022, e02819. doi: 10.1111/jav.02819

[B73] Soares L. Ellis V. A. Ricklefs R. E. (2016a). Co-infections of haemosporidian and trypanosome parasites in a North American songbird. Parasitology 143, 1930–1938. doi: 10.1017/S0031182016001384, PMID: 27644582

[B74] Soares L. Escudero G. Penha V. A. S. Ricklefs R. E. (2016b). Low prevalence of haemosporidian parasites in shorebirds. Ardea 104, 129–141. doi: 10.5253/arde.v104i2.a8

[B75] Stenkat J. Krautwald-Junghanns M.-E. Schmidt V. (2013). Causes of morbidity and mortality in free-living birds in an urban environment in Germany. Ecohealth 10, 352–365. doi: 10.1007/s10393-013-0868-9, PMID: 24136384

[B76] Tchoumbou M. A. Mayi M. P. A. Malange E. N. F. Foncha F. D. Kowo C. Fru-cho J.. (2020). Effect of deforestation on prevalence of avian haemosporidian parasites and mosquito abundance in a tropical rainforest of Cameroon. Int. J. Parasitol. 50, 63–73. doi: 10.1016/j.ijpara.2019.10.006, PMID: 31866311

[B77] Thomas R. C. Dunn J. C. Dawson D. A. Hipperson H. Horsburgh G. J. Morris A. J.. (2022). Assessing rates of parasite coinfection and spatiotemporal strain variation via metabarcoding: Insights for the conservation of European turtle doves *Streptopelia turtur* . Mol. Ecol. 31, 2730–2751. doi: 10.1111/mec.16421, PMID: 35253301 PMC9325524

[B78] Tobler M. W. Kéry M. Hui F. K. C. Guillera-Arroita G. Knaus P. Sattler T.. (2019). Joint species distribution models with species correlations and imperfect detection. Ecology 100, e02754. doi: 10.1002/ecy.2754, PMID: 31062356

[B79] Townsend A. K. Clark A. B. McGowan K. J. Miller A. D. Buckles E. L. (2010). Condition, innate immunity and disease mortality of inbred crows. Proc. R. Soc. B: Biol. Sci. 277, 2875–2883. doi: 10.1098/rspb.2010.0480, PMID: 20444716 PMC2981987

[B80] Tsiodras S. Kelesidis T. Kelesidis I. Bauchinger U. Falagas M. E. (2008). Human infections associated with wild birds. J. Infection 56, 83–98. doi: 10.1016/j.jinf.2007.11.001, PMID: 18096237 PMC7172416

[B81] Valkiunas G. (2005). Avian malaria parasites and other haemosporidia (New York: CRC Press).

[B82] Verwey J. Peters A. Monks D. Raidal S. (2018). Spillover of avian haemosporidian parasites (Haemosporidia: *Plasmodium*) and death of captive psittacine species. Aust. Vet. J. 96, 93–97. doi: 10.1111/avj.12671, PMID: 29479679

[B83] Von-Matter S. Straube F. C. Accordi I. A. Piacentini V. Q. Cândido-Jr J. F. (2010). Ornitologia e Conservação: Ciência aplicada, técnicas de pesquisa e levantamento (Rio de Janeiro: Technical Books Editora).

[B84] Wilson K. Bjørnstad O. N. Dobson A. P. Merler S. Poglayen G. Randolph S. E.. (2002). “Heterogeneities in macroparasite infections: patterns and processes,” in The ecology of wildlife diseases, eds. Hudson P. J. Rizzoli A. Grenfell B. T. Heesterbeek H. Dobson A. P., (Oxford, United Kingdom: Oxford University Press Oxford), 6–44. doi: 10.1093/oso/9780198506201.003.0002

[B85] Wojczulanis-Jakubas K. Jakubas D. Czujkowska A. Kulaszewicz I. Kruszewicz A. G. (2012). Blood parasite infestation and the leukocyte profiles in adult and immature reed warblers (*Acrocephalus scirpaceus*) and sedge warblers (*Acrocephalus schoenobaenus*) during autumn migration. Ann. Zool Fennici 49, 341–349. doi: 10.5735/086.049.0507

[B86] Woolhouse M. E. J. Webster J. P. Domingo E. Charlesworth B. Levin B. R. (2002). Biological and biomedical implications of the co-evolution of pathogens and their hosts. Nat. Genet. 32, 569–577. doi: 10.1038/ng1202-569, PMID: 12457190

[B87] Yoshimoto M. Ozawa K. Kondo H. Echigoya Y. Shibuya H. Sato Y.. (2021). A fatal case of a captive snowy owl (*Bubo scandiacus*) with *Haemoproteus* infection in Japan. Parasitol. Res. 120, 277–288. doi: 10.1007/s00436-020-06972-1, PMID: 33191448

