# Supplementary material for: Physiological, environmental, and life-history drivers of haemosporidian infections in non-passerine birds from a rehabilitation center
Source: Front Parasitol. 2025 Aug 4;4:1568852. doi: 10.3389/fpara.2025.1568852 (PMC12358355; doi:10.3389/fpara.2025.1568852)
Supplement: Supplementary file 1 [file Table1.docx]

*Online Supplementary Materials*

Supplementary Table 1: Summary of the top models, including degrees of freedom, delta AIC (∆AIC), and weights (wi), for predicting the occurrence of combined haemosporidian parasites (*Plasmodium* spp. and *Haemoproteus* spp. together), of *Plasmodium* spp. alone, of *Haemoproteus* spp. alone, and haemosporidian severity, using four different generalized linear mixed models (GLMM). Predictors included microhematocrit levels, age, season, H/L ratio, body condition, and presence/absence of major injuries, trichomoniasis, and ticks. To account for potential phylogenetic relatedness among avian hosts, the host species' binomial name was included as a random effect in models. All models included age and season as fixed effects. To ensure model convergence and stability, individuals positive for *Trichomonas* (n = 5) were excluded. We present only models with a weight (w_i_) less than two, and lower than one for the severity model (due to the high number of different models).

| Models | df | ∆AIC | w_i_ |
| --- | --- | --- | --- |
| **Response variable: Haemosporidian parasites occurrence (both *Plasmodium* spp. and *Haemoproteus* spp. together)** |  |  |  |
| Age | 3 | 0.00 | 0.061 |
| Age + Body condition | 4 | 0.19 | 0.056 |
| Age + Season | 4 | 0.34 | 0.052 |
| Age + Body condition + Season | 5 | 1.32 | 0.052 |
| Age + Microhematocrit level | 4 | 1.41 | 0.030 |
| Age + Season + H/L ratio | 5 | 1.78 | 0.025 |
| Age + *Trichomonas* spp. occurrence | 4 | 1.81 | 0.025 |
| Age + Season + Microhematocrit level | 5 | 1.82 | 0.0.25 |
| Age + H/L ratio | 4 | 1.98 | 0.023 |
|  |  |  |  |
| **Response variable: *Plasmodium* spp. occurrence** |  |  |  |
| Season | 3 | 0.62 | 0.056 |
| Age | 3 | 1.43 | 0.038 |
| Body condition | 3 | 1.83 | 0.031 |
| Microhematocrit level | 3 | 1.87 | 0.030 |
| *Trichomonas* spp. occurrence | 3 | 1.93 | 0.029 |
| Major injuries | 3 | 1.96 | 0.029 |
|  |  |  |  |
| **Response variable: *Haemoproteus* spp. occurrence** |  |  |  |
| Age + Season + H/L ratio | 5 | 0.00 | 0.165 |
| Age + Season + Microhematocrit level + H/L ratio | 6 | 1.34 | 0.084 |
| Age + Season + *Trichomona*s spp. occurrence + H/L ratio | 6 | 1.65 | 0.072 |
|  |  |  |  |
| **Response variable: Haemosporidian parasite severity** |  |  |  |
| Age + Tick occurrence | 5 | 0.00 | 0.034 |
| Tick occurrence | 4 | 0.25 | 0.030 |
| Tick occurrence + *Trichomonas* spp. occurrence | 5 | 0.64 | 0.025 |
| Age + Tick occurrence + *Trichomonas* spp. occurrence | 6 | 0.76 | 0.023 |
| Age + Tick occurrence + Season | 6 | 0.94 | 0.021 |
| Age | 4 | 0.94 | 0.021 |
| Age + Tick occurrence + Major injuries | 6 | 0.95 | 0.021 |
| Age + Major injuries | 5 | 0.99 | 0.021 |

Supplementary Table 2: Predictors of total haemosporidian infection (*Plasmodium* + *Haemoproteus* together). Here and below, C.I. = confidence interval.

| Predictor | Estimate | Std. Error | p-value | 2.5 C.I. | 97.5 C. I. |
| --- | --- | --- | --- | --- | --- |
| Intercept | -0.1453 | 0.743 | 0.8475 | -1.6266 | 1.3359 |
| Age | -1.4149 | 0.7342 | 0.0588 | -2.8825 | 0.0526 |
| HL | 0.5685 | 0.3618 | 0.1232 | -0.1542 | 1.2913 |
| Season | 0.7433 | 0.7797 | 0.3498 | -0.8147 | 2.3012 |
| Condition | -0.6953 | 0.8018 | 0.3949 | -2.2969 | 0.9063 |
| Microhematocrit | 0.3694 | 0.3788 | 0.3385 | -0.3871 | 1.1258 |
| Major lesions | 0.7488 | 0.728 | 0.3127 | -0.705 | 2.2026 |

Supplementary Table 3: Predictors of *Plasmodium* infection.

| Predictor | Estimate | Std. Error | p-value | 2.5 % | 97.5 % |
| --- | --- | --- | --- | --- | --- |
| Intercept | -1.7736 | 0.6933 | 0.012 | -3.1567 | -0.3906 |
| Season | -1.1416 | 0.7217 | 0.121 | -2.5855 | 0.3024 |
| Major lesions | 0.9871 | 0.6797 | 0.155 | -0.3721 | 2.3462 |
| Microhematocrit | 0.443 | 0.3268 | 0.184 | -0.2111 | 1.0971 |
| Condition | -0.5833 | 0.7938 | 0.471 | -2.1681 | 1.0015 |
| Age | -0.1711 | 0.6627 | 0.8 | -1.4955 | 1.1533 |
| HL | -0.0729 | 0.3418 | 0.834 | -0.756 | 0.6101 |

Supplementary Table 4: Predictors of *Haemoproteus* infection.

| Predictor | Estimate | Std. Error | p-value | 2.5 C.I. | 97.5 C.I. |
| --- | --- | --- | --- | --- | --- |
| Intercept | -3.0609 | 1.566 | 0.0551 | -6.1892 | 0.0674 |
| Age | -3.3126 | 1.6813 | 0.0537 | -6.6781 | 0.053 |
| Season | 3.9333 | 1.9756 | 0.0505 | -0.009 | 7.8756 |
| HL | 1.3635 | 0.6658 | 0.0448 | 0.0319 | 2.695 |
| Microhematocrit | 0.6532 | 0.6175 | 0.3003 | -0.5828 | 1.8892 |
| Condition | -0.0954 | 1.3353 | 0.9442 | -2.7665 | 2.5757 |
| Major lesions | -0.2804 | 1.0894 | 0.8012 | -2.4624 | 1.9016 |

Supplementary Table 5: Number of individuals sampled per species, season, and age class. This table summarizes the number of individuals per species classified by season (Dry/Rainy) and age (Adult/Juvenile).

| Species | Dry_Adult | Rainy_Adult | Dry_Juvenile | Rainy_Juvenile |
| --- | --- | --- | --- | --- |
| *Aramides cajanea* | 4 | 3 | 0 | 0 |
| *Caracara plancus* | 12 | 1 | 13 | 2 |
| *Cariama cristata* | 7 | 3 | 2 | 1 |
| *Megascops choliba* | 6 | 2 | 4 | 5 |
| *Milvago chimachima* | 3 | 1 | 1 | 0 |
| *Penelope obscura* | 0 | 0 | 2 | 3 |
